# Exploring pH Dynamics in Amino Acid Solutions Under Low-Temperature Plasma Exposure

**DOI:** 10.3390/molecules29245889

**Published:** 2024-12-13

**Authors:** Cecilia Julieta Garcia Villavicencio, Beatriz de Campos Silva, Anesu Matara, Sylwia Ptasinska

**Affiliations:** 1 Radiation Laboratory, University of Notre Dame, Notre Dame, IN 46556, USA; cgarcavi@nd.edu (C.J.G.V.); bdecampo@alumni.nd.edu (B.d.C.S.); amatara@nd.edu (A.M.); 2 Department of Physics and Astronomy, University of Notre Dame, Notre Dame, IN 46556, USA

**Keywords:** low-temperature plasma, amino acids, pH, reactive oxygen and nitrogen species, infrared spectroscopy

## Abstract

Low-temperature plasma (LTP) offers a promising alternative for cancer therapy, as it targets malignant cells selectively while minimizing damage to healthy tissues. Upon interaction with an aqueous solution, LTP generates reactive oxygen and nitrogen species and thereby influences the solution’s pH, which is a crucial factor in cancer proliferation and response to treatment. This study investigated the effects of LTP on the pH of aqueous solutions, with a focus on the effect of LTP parameters such as voltage, frequency, and irradiation time. In addition, it explored the influence of solution composition, specifically the presence of the amino acids, glycine and serine, on pH changes; these amino acids are known to play significant roles in cancer proliferation. Our results indicated that LTP induces acidification in deionized water, in which the extent of acidification increased proportionally with plasma parameters. In glycine-containing solutions, pH changes were concentration-dependent, whereas serine-containing solutions maintained a constant pH across all tested concentrations. To investigate potential changes to the structural properties of glycine and serine exposed to LTP that could be responsible for different pH responses, we analyzed the samples using FTIR spectroscopy. A significant decrease in absorbance was observed for solutions with low concentrations of amino acids, suggesting their degradation.

## 1. Introduction

Low-temperature plasma (LTP) has been used in a wide variety of applied research and industrial areas and has recently facilitated important medical advancements. Indeed, LTP has been employed broadly for biomedical applications [1], such as wound healing [2,3], sterilization [3,4,5], tooth treatment [6,7], blood coagulation [8,9], pharmaceuticals [10], and cancer therapies, having been demonstrated to effectively kill tumor cells without damaging surrounding tissues [10,11]. Moreover, LTP inhibits the proliferation, metastasis, and invasion of various cancer cells alongside inducing apoptosis [12,13,14,15,16]. With the emergence of the plasma medical field, mainly plasma oncology, many recent reviews have focused on the biological influence of plasmas, particularly their lethal and selective effects on cancer cells [17,18,19,20]. Beyond these promising results in cancer treatment, however, there remain questions regarding the underlying mechanisms and aspects responsible for such effects.

Among several potential factors, changes in the pH of plasma-irradiated tissue can be a crucial contributor to the effects of LTP on cancer cells. That is, pH is important in cancer cell growth, division, and spread as it influences most cellular processes and microenvironments [21,22,23]. In particular, a low extracellular pH enhances tumor drug resistance and promotes invasive growth and metastasis. The acidic nature of cancer cells has led some to suggest using an alkaline (basic) pH treatment to restore the normal pH gradient as a complement to existing therapies [24,25]. However, Zimmermann et al. [26] reported that acidified nitrite is a potent inhibitor of melanoma cells; in addition, the acidic conditions in tumors could support the effects of LTP and contribute to the treatment’s cancer cell specificity [27].

Another critical factor in cancer proliferation is the presence of amino acids, essential nutrition and energy sources that promote and participate in pathways that feed cancer cells [28]. In particular, amino acids can influence reactive oxygen species (ROS) homeostasis and epigenetic regulation by methylation and acetylation, which can in turn intensify tumor aggressiveness [29]. A number of processes targeting amino acid metabolism have been applied in the interest of developing better therapeutic strategies [30].

Given the importance of these factors, a systematic investigation of the pH level in solutions with and without amino acids under a wide range of plasma treatment conditions can offer an essential foundation for developing and refining strategies for targeting cancer cells and their metabolism. It is well known that plasma irradiation induces a decrease in the pH of the treated system, which is attributed principally to the production of acid species such as nitrous and nitric acids [31,32] that contribute to more protons (H^+^) in the solution. The degree of acidification depends on LTP parameters, such as frequency, gas flow rate, and irradiation time, and also on the buffer capacity of the medium. Heuer et al. [33] previously reported significant acidification when the skin is treated with LTP, and Busco et al. [34] performed studies with agarose gel models to observe and quantify acidification of the tissue surface.

In addition, several reports have described LTP interaction with amino acids. For example, LTP irradiation of proline and hydroxyproline has been shown to result in oxidative modifications such as the formation of C–OH and C=O groups and cleavage of the pyrrolidine ring [35]. The oxidation of side chains by LTP was also reported for 14 amino acids, attributed to the production of and reaction with reactive oxygen and nitrogen species (RONS) [36]. Aromatic (phenylalanine, tyrosine, and tryptophan) and sulfur-containing (cysteine and methionine) amino acids demonstrated the highest reactivity with RONS. In addition to oxidation, LTP irradiation of valine and alanine caused degradation of the -COOH and -NH_2_ groups, which resulted in the decomposition of these amino acids into various products, such as acetone, formic acid, acetic acid, aspartic acid, threomethyl, pyruvate, and red methyl aspartic acid [37,38]. As LTP can have a number of effects on amino acids, understanding their interactions is essential to the development of better strategies for biomedical applications.

In this study, we aim to investigate the effects of plasma irradiation on solution pH in relation to various plasma parametric conditions. First, we investigated the LTP effects on deionized (DI) water; then, we diluted each of the two amino acids in DI water and observed the impact of each biomolecule on the pH of the irradiated solution. The amino acids selected were glycine (Gly) and L-serine (Ser), the biosynthesis of which affects cellular oxidative capacity and supports tumor homeostasis [39,40]. In addition, we performed Fourier transform infrared (FTIR) spectroscopy on the irradiated samples to study any potential LTP-induced changes in glycine and serine. Alterations to these amino acids can lead to the inhibition of their metabolism and therefore might improve tumor control as part of a treatment plan [41]. Ultimately, understanding how plasma treatment affects solution pH can help develop better strategies for using LTP in cancer treatment and facilitate the prediction of possible collateral effects on surrounding tissues and microenvironments that are strongly influenced by extracellular pH.

## 2. Results

We performed experiments with DI water and with solutions containing glycine and serine to determine how the presence of these amino acids influences pH changes under LTP irradiation. In addition, we observed the effect of increasing voltage and frequency on solution pH.

### 2.1. pH Measurements of Irradiated DI Water

We conducted the initial set of experiments using DI water, maintaining a constant voltage of 8 kV while varying the frequency within the range of 1–4 kHz. For the second set, we increased the voltage to 10 kV while retaining the same frequency range.

**Figure 1:**
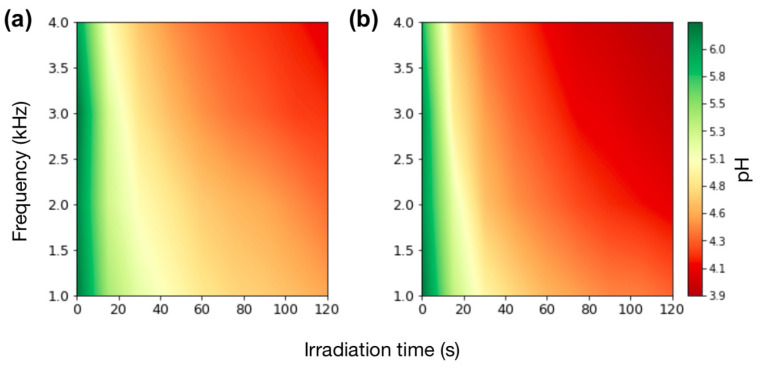
Solution pH after irradiation of DI water using different plasma parameters at a constant voltage of 8 kV (**a**) and 10 kV (**b**).

In Figure 1, changes in the pH of irradiated solutions are visualized using a colormap. Moderately acidic pH values are shown in orange and red hues, corresponding to a pH range of 4.8–3.9, while weakly acidic pH values are illustrated with green and yellow, corresponding to a pH range of 6.0–5.1. At both of the tested voltage levels, the final solution pH depended on frequency and irradiation time.

Figure 1a presents the results obtained with a voltage of 8 kV while changing the frequencies and irradiation time. We observed pH values from 6.3 to 4.6 at 1 kHz and from 6.0 to 4.1 at 4 kHz. The lowest pH values were obtained with the longest irradiation time of 120 s, resulting in 4.6, 4.4, 4.3, and 4.1 for frequencies of 1, 2, 3, and 4 kHz, respectively. Overall, longer treatment times and higher frequencies resulted in more acidic pH values.

Similarly, Figure 1b illustrates the results for a voltage of 10 kV while changing the frequencies and irradiation time. When using a frequency of 1 kHz, the resultant pH values ranged from 6.3 to 4.4, and for 4 kHz, values ranged from 5.9 to 3.9. Similar to the previous parameter set, the lowest values were observed after 120 s of irradiation, producing solutions with a respective pH of 4.4, 4.1, 4.0, and 3.9 for frequencies of 1, 2, 3, and 4 kHz. Comparing the results for 8 kV and 10 kV, it is evident that acidic values are more pronounced under higher voltage, indicating that greater voltage enhances the predominance of acidic pH.

### 2.2. pH Measurements of Irradiated Amino Acid Solutions

Subsequently, we carried out experiments using glycine and serine solutions under the same irradiation conditions for irradiation times ranging from 0 to 120 s, only employing two extreme conditions of plasma parameters: 8 kV at 1 kHz and 10 kV at 4 kHz, corresponding to the average instantaneous power per pulse of 2.9 and 24 W.

**Figure 2:**
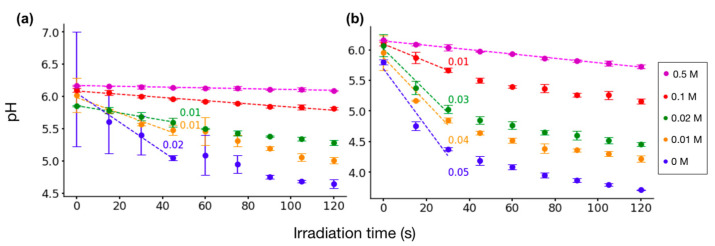
pH evolution of glycine solutions at different concentrations between 0.01–0.5 M and DI water (0 M) as a function of irradiation time and plasma parameters: (**a**) 8 kV and 1 kHz; (**b**) 10 kV and 4 kHz. Error bars represent the standard deviation of three individual experiments performed for each irradiation duration. Values represent the rate of change in pH for the linear region of the data for changes higher than 0.01 pH per second.

Figure 2 illustrates the pH changes in irradiated glycine solutions for both plasma conditions. Figure 2a presents the trends with values for the rate of change in pH calculated from the linear fit of the experimental data recorded at 8 kV and 1 kHz.

A stable pH was evident for the 0.5 M solution, whereas the 0.1 M solution exhibited a noticeable decrease in pH. Meanwhile, the lower concentrations of 0.02 M and 0.01 M both showed a relatively significant decrease in pH at the rate of 0.01 units per second over the first 30 s of treatment.

Figure 2b presents glycine solution pH changes under the second set of parameters, 10 kV and 4 kHz. Decreases in pH were more pronounced at this higher voltage and frequency. Again, the highest concentration (0.5 M) exhibited minimal effect. For the lower concentrations of 0.02 M and 0.01 M, the rate of change during the first 30 s was 0.03 and 0.04 units per second, respectively. After that initial period, the rate transitioned to a less pronounced decline.

These observations indicate that higher plasma energy input (10 kV and 4 kHz) results in a more substantial acidification effect, particularly at lower glycine concentrations. The highest concentration tested (0.5 M) was more resistant to pH change. These results underscore the importance of both plasma parameters and solute concentration in determining the extent of pH change following plasma irradiation.

**Figure 3:**
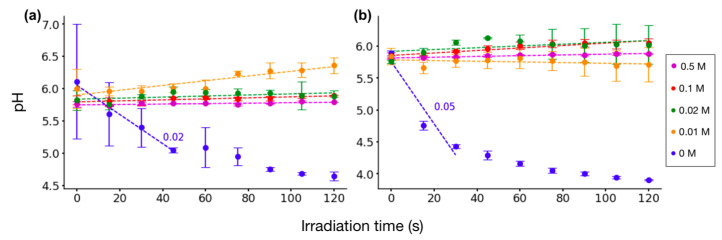
pH evolution of serine solutions at different concentrations between 0.01–0.5 M and DI water (0 M) as a function of irradiation time and plasma parameters: (**a**) 8 kV and 1 kHz; (**b**) 10 kV and 4 kHz. Error bars represent the standard deviation of three individual experiments performed for each irradiation duration. Values represent the rate of change in pH for the linear region of the data for changes higher than 0.01 pH per second.

For serine solutions, the pH remained relatively stable under both sets of plasma parameters. As shown in Figure 3a, irradiation of the 0.01 M solution at 8 kV and 1 kHz resulted in a slight increase in pH to 6.1. For the highest concentration of 0.5 M, the pH remained relatively constant at around 5.7. Similar results were observed with higher plasma parameters, as illustrated in Figure 3b.

The duration of irradiation was a significant factor influencing pH. For glycine solutions, the pH decreased with increasing irradiation time, and the lowest pH values were consistently observed following 120 s of irradiation (5.7, 5.2, 4.7, and 4.2 for concentrations of 0.5 M, 0.1 M, 0.02 M, and 0.01 M, respectively). For serine solutions, a slight increase in pH was evident with prolonged irradiation.

Thus, our results indicate that for glycine solutions, pH becomes more acidic with longer irradiation, and this decrease is concentration-dependent, whereas for serine solutions, the pH remains closer to near-neutral acidic, with a slight increase as irradiation time increases.

### 2.3. FTIR Spectroscopy of Irradiated Amino Acid Solutions

We used FTIR spectroscopy to characterize any potential chemical and molecular changes in our samples after plasma treatment. We analyzed the infrared spectra for each amino acid with two different concentrations (0.01 M and 0.5 M) without LTP irradiation and irradiated at 10 kV and 4 kHz for 120 s.

**Figure 4:**
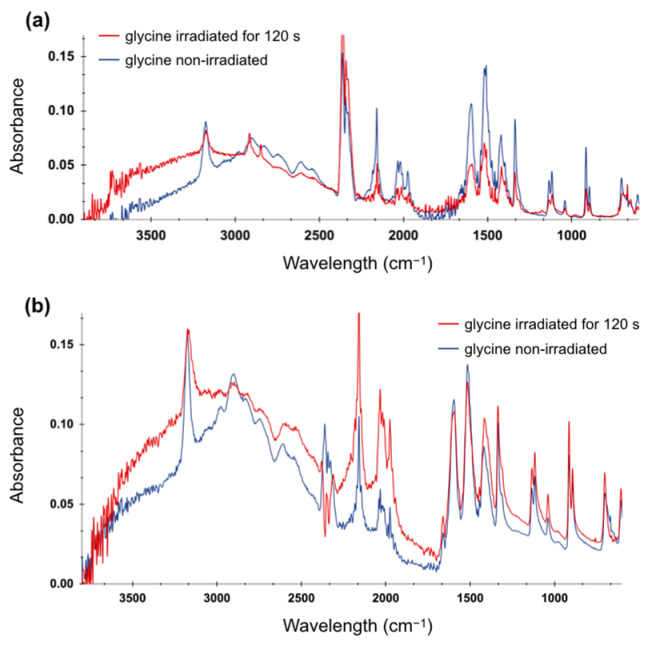
FTIR spectra of glycine irradiated for 120 s at 10 kV and 4 kHz and non-irradiated: (**a**) with a concentration of 0.01 M, (**b**) with a concentration of 0.5 M.

Figure 4a presents the spectra of non-irradiated glycine and that following 120 s of irradiation for the lower concentration. We identified the characteristic features in the glycine spectra based on previous reports of crystal glycine [42,43] and glycine dissolved in water [44]. For example, the symmetrical N-H stretching band appears as a sharp peak at 3170 cm^−1^, consistent with the amine group vibrations, while the 2900 cm^−1^ peak represents a C-H stretching. The peaks at 1597, 1516, and 1415 cm^−1^, which can be attributed to the NH_2_ bending from the amino group and the peak at 1333 cm^−1^ corresponding to the mixture of CH_2_ and N-H bend, showed a decrease in intensity [44]. We did not observe any emergence of new peaks in the irradiated sample, indicating that plasma did not create any new bonds or functional groups. However, we noted a decrease in absorbance, suggesting that plasma treatment reduced the concentration of glycine due to its potential degradation.

In the case of irradiated glycine with a higher concentration (0.5 M), we observed less pronounced changes, as shown in Figure 4b. This suggests that LTP treatment does not have a significant impact on solutions containing higher concentrations of the amino acid.

**Figure 5:**
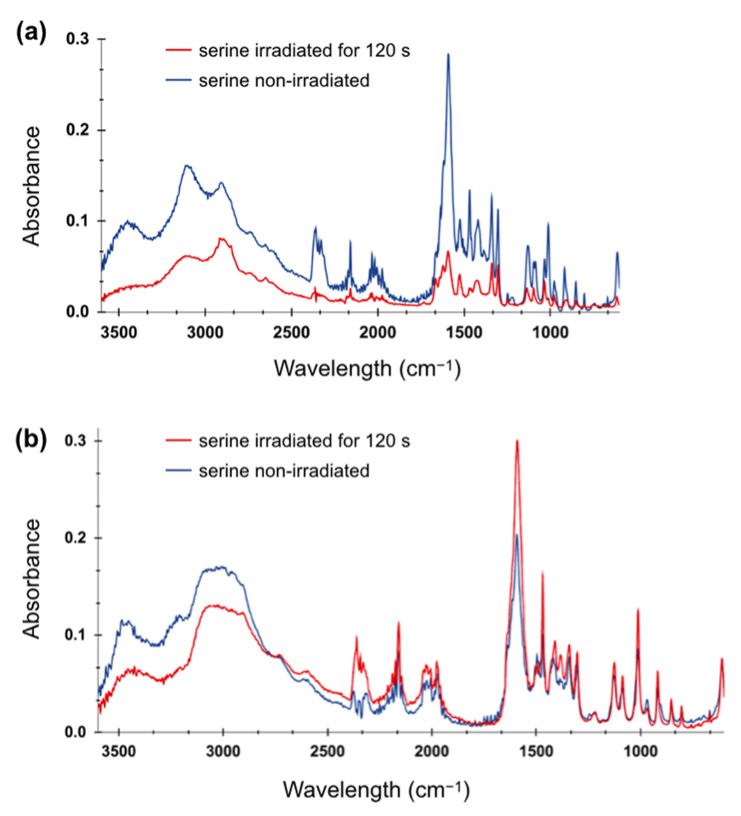
FTIR spectra of serine irradiated for 120 s at 10 kV and 4 kHz and non-irradiated: (**a**) with a concentration of 0.01 M, (**b**) with a concentration of 0.5 M.

Similar to glycine, the plasma irradiation of serine solutions with concentrations of 0.01 M and 0.5 M did not lead to the formation of new bonds or functional groups, but a decrease in the absorbances of certain peaks was observed (Figure 5). For example, there is a significant decrease in the absorbance of the OH and NH₃^+^ stretching bands [45] in the 3500–3000 cm^−1^ region. In addition, the decrease in absorbance is evident for serine at 0.01 M, in which the NH₃⁺ asymmetric bending band (1593 cm^−1^) [46] showed significant reduction, suggesting possible degradation of serine. Plasma irradiation appears to play a critical role in the loss of the molecular structure of serine, particularly at this low concentration, as observed in Figure 5a. In the case of serine at 0.5 M, as shown in Figure 5b, the irradiated sample exhibits less change. While there was a noticeable reduction in absorbance in the O-H and N-H stretching region (between 3500–3000 cm^−1^), the fingerprint region does not exhibit significant changes. This suggests that at higher concentrations of serine, LTP does not induce sample degradation.

For both glycine and serine, we observed some features in the 2500–2000 cm^−^^1^ region, which can be attributed to the overtone and combination bands of both amino acids [44,45], as well as the peaks at 2300 cm^−^^1^, which can be attributed to CO_2_ [47]. Importantly, because amino acids are dissolved in water, water can exhibit bands in a spectrum with a broad peak at around 3400 cm^−^^1^, corresponding to the stretching vibrations of OH, and a sharp feature at 1640 cm^−^^1^, corresponding to the bending vibration of HOH [48]. Therefore, both features can interfere with features from amino acids, which is especially observable in the case of the serine spectra.

## 3. Discussion

In this study, we investigated how plasma parameters can affect the final pH of an irradiated solution. Specifically, we applied plasma to DI water and aqueous solutions of amino acids (glycine and serine). We observed induced acidification in irradiated DI water samples and in solutions containing lower concentrations of glycine. Greater voltage and frequency enhanced LTP-induced acidification, especially greater voltage; in addition, we observed the lowest pH at the longest irradiation time of 120 s. These results align with those of several prior reports of LTP-induced acidification of water [32,49,50,51,52].

Moreover, the acidification of DI water and glycine (0.01 M and 0.02 M) was rapid, occurring within 30 s of irradiation. Meanwhile, solutions with higher concentrations of glycine (0.5 M and 0.1 M) or any amount of serine demonstrated a slower rate of pH change or no change throughout the irradiation period, indicating that a sufficient quantity of these amino acids may buffer the solution, mitigating the extent of acidification compared to DI water. The acidification of water following LTP exposure is mainly attributed to RONS delivered from the LTP source. Several factors affect RONS type and concentration, namely the feed gas and other plasma parameters, the type of plasma source, and ambient conditions [53]. The species most commonly reported as being formed in aqueous solutions during LTP irradiation include hydrogen peroxides, nitrates, nitrites, superoxide anions, ozone, nitric oxide radicals, and hydroxyl radicals [49,53]. These species exhibit a wide range of lifetimes; some are relatively long-lived, while others are much more transient. This is because their reactivity is closely related to their lifetimes, which in turn influences their interactions with the irradiated systems. The continuous generation of RONS during plasma interactions means that the overall composition of reactive species in the solution can change dynamically throughout the irradiation. As a result, irradiation time becomes a crucial factor in determining the final outcome. Prolonged exposure may lead to a buildup of long-lived species, which can persist in the solution for extended periods and increase the likelihood of secondary reactions. While shorter exposures might be dominated by the effects of the short-lived species, highly reactive species tend to exert their effects almost immediately upon formation. In our previous study using the same LTP source, we quantified short-lived plasma species that are OH radicals [54] and solvated electrons [55], which are known to be major players in initiating reactions in the solution during plasma irradiation [56]. Specifically, we determined the rates of change in the concentration of both species to be nearly equivalent; that is, in the case of OH radicals, 0.45 and 2 μM s^−1^, and in the case of electrons, 1.25 and 2.5 μM s^−1^ for 8 kV and 1 kHz and 10 kV and 4 kHz conditions, respectively [55].

Indeed, in our studies, we identified two distinct regions with different rates of pH change for LTP-irradiated DI water. The first region, occurring within the first 30 s, showed a rapid decrease in pH. Specifically, under 8 kV and 1 kHz, the initial rate of change was 0.02 pH units per second, while for 10 kV and 4 kHz, the rate was 0.05 pH units per second. The second region observed for irradiation longer than 30 s showed a slower decrease in pH, with the rate being 0.01 pH units per second for both conditions. This suggests different chemical compositions in both regions involving short- and long-lived species, respectively, as well as secondary products in the second region.

Furthermore, the medium that is irradiated is another important factor in determining the final solution pH. A previous study [57] analyzed the RONS generated by LTP in three different media and found medium composition to have a major influence on solution pH during plasma treatment, the stability of the various RONS, and RONS reactivity with biomolecules. When amino acids are present in plasma-irradiated water, they may be modified by reactive species. One study [36] examined side chain oxidization in fourteen amino acids and determined these chemical modifications to be caused by interactions with RONS and not by acidic pH. Although, as reported by other studies, amino acid degradation is mainly attributed to interactions with RONS, low solution pH could also contribute to the chemical degradation of amino acids [58].

In our study, the two amino acid solutions tested responded differently to LTP irradiation, showing different trends for changes of solution pH that might be explained by degradation or changes in amino acid forms. Glycine, the simplest amino acid, presents a pH-dependent polymorphism that results in multiple forms in water: cation (Gly^+^), zwitterion (Gly^±^), and anion (Gly^−^) [59]. Glycine molecules form open, hydrogen-bonded dimers in water occurring between the pH range of 4 to 8. Beyond this range, the monomeric zwitterions can be facilitated by charged species in the solution [59]. In the case of lower pH, at a pH below 2.35 (pKa₁), the formation of an ionized amino group, i.e., -NH_3_^+^, occurs with a neutral carboxyl group [59]. At physiological pH (~7), glycine exists in its zwitterionic form, where the amino group is protonated (NH₃⁺) and the carboxyl group is deprotonated (COO⁻). Above pH 9.78 (pKa₂), glycine loses a proton from the amino group, becoming negatively charged overall. Because LTP significantly decreases pH, but not below glycine’s pKa₁, the zwitterionic form will still dominate, but there might be an increase in the ratio of protonated species from the increase in H⁺ ions.

Notably, the change in pH also depends on the molar concentration of glycine, as higher concentrations may maintain a constant pH. In contrast, at low concentrations of glycine, the LTP-induced acidification affects glycine’s buffering capacity from the smaller amount of carboxyl groups in comparison to the excess H⁺ ions that can lower pH. We reported previously that H⁺ ion concentration increases with irradiation time, and their formation yields are ~0.2 mM s^−1^ and 1.4 mM s^−1^ for 8 kV and 1 kHz and 10 kV and 4 kHz conditions, respectively. When the amino acid concentration is high, the carboxyl groups may not all become protonated, and the solution therefore maintains the same pH.

Meanwhile, LTP treatment of serine solutions did not significantly alter pH, which remained weakly acidic. This contrasts with the acidification observed in deionized water and glycine-containing solutions under similar conditions. In the case of serine, its pKa values are slightly lower than those of glycine, but its side-chain hydroxyl group has a high pKa (~13), and the LTP treatment did not directly deprotonate the hydroxyl group.

Most of the identified features in the FTIR spectra are characteristics of zwitterion groups of both amino acids [44,45]. These spectral features significantly decreased after LTP irradiation in the samples with low concentrations that can identify the change in the amino acid form or sample degradation. Whereas, for the higher-concentration solutions, these features were preserved, indicating that most molecules are physically shielding each other, thus minimizing the effect of plasma irradiation. As mentioned above, no new features from the formation of new functional groups or chemical bonds were observed in the irradiated samples, confirming the results of other studies in which chemical modifications after plasma exposure, more specifically the oxidation of a few amino acids, including glycine and serine, were not found [36].

Moreover, the pH stability of serine solutions can be attributed to the structural and chemical properties of the amino acid, particularly its interactions with water or, in general, with a polar solvent. While both amino acids have polar groups, the side chain hydroxyl group (-OH) in serine can form hydrogen bonds. The absence of such additional functional groups in glycine can result in weaker interactions with water molecules and potentially with RONS. In addition, glycine is hydrophobic, whereas serine is hydrophilic; consequently, serine has a stronger interaction with water molecules than glycine does. A stronger interaction of water with serine can be confirmed with the obtained infrared spectra, which showed a stronger contribution of water-characteristic features in serine but a negligible one for glycine.

Further research using other analytical techniques should explore the detailed mechanisms by which serine, glycine, and other polar and nonpolar amino acids interact with RONS and become degraded. Elucidating the behavior of amino acids with their different polarities can provide a broader understanding of how LTP can be optimized for therapeutic applications. Additionally, examining pH changes in the living cell upon irradiation may inform strategies not only for optimizing but also for mitigating side effects that LTP treatment might exert on essential protein structures, DNA, and other cellular components.

## 4. Materials and Methods

### 4.1. Plasma Setup

**Figure 6:**
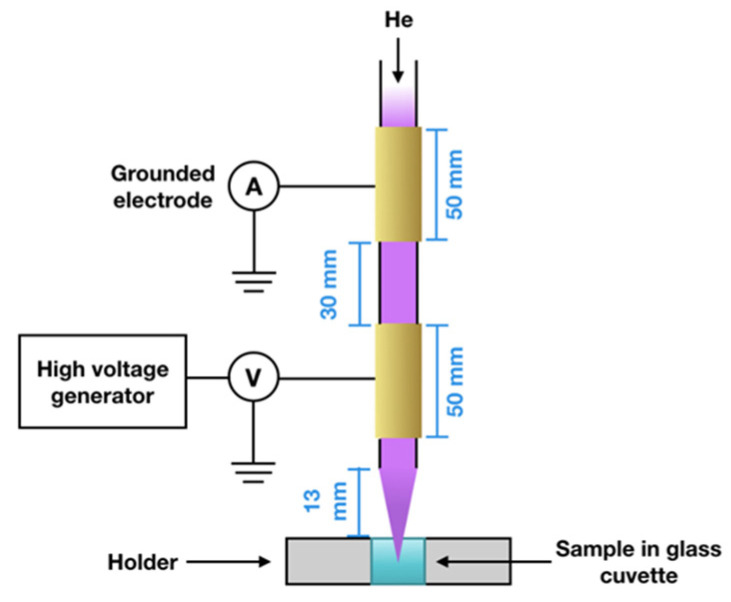
Schematic of the experimental setup.

A diagram of the helium-discharge plasma source used to irradiate the samples is shown in Figure 6, and has been described previously [60]. Briefly, the source contains two brass electrodes of 50 mm in length, one connected to a high-voltage power generator and the other grounded. The electrodes are mounted outside a fused silica capillary with a 5 mm diameter. Ultra-high-purity He was used as the feed gas, with the flow rate maintained at 2 standard liters per minute (slm) by a flow rate controller from Bronkhorst High-Tech, Bethlehem, PA, USA. When high-voltage pulses are applied to the electrode, plasma is ignited in the fused silica capillary and launched into the air, forming a plasma jet of 3 cm in length. For irradiation, voltages of 8 kV and 10 kV and frequencies in the range of 1–4 kHz were chosen. This combination of plasma parameters produces a jet with a measured temperature below 80 °C [61].

We estimated the average instantaneous plasma power per pulse from the current-voltage characteristics of the plasma source as reported in [61,62]. The power increased when the frequency or voltage was increased, so the power per pulse for frequencies from 1 to 4 kHz was 2.9, 5.9, 8.9, and 11.9 W for 8 kV and 6, 12, 18, and 24 W for 10 kV.

Because LTPs are generated in open-air conditions, differences in humidity can affect the properties of the plasma discharge [63]. Therefore, the humidity in the laboratory was monitored using a humidity sensor from ThermoPro, Sugar Land, TX, USA. The recorded humidity was in the range of 42% to 51% for all irradiation sets.

### 4.2. Solution Irradiation

For DI water irradiation, 500 μL of water in a glass cuvette was secured beneath the plasma jet to ensure proper contact of the plasma with the sample surface, and the solution was exposed to plasma irradiation for a time interval ranging from 0 to 120 s. Three trials per each plasma condition were performed.

Glycine and L-serine were purchased from Sigma Aldrich, St. Louis, MO, USA. Amino acid solutions were prepared at concentrations of 0.5 M, 0.1 M, 0.02 M, and 0.01 M. Samples of each concentration were irradiated under two extreme combinations of voltage and frequency: 8 kV and 1 kHz, and 10 kV and 4 kHz.

### 4.3. pH Measurement

A pH meter, Orion Versa Star from Thermo Fisher Scientific, Waltham, MA, USA, with an accuracy of ±0.002, and an Orion Semi-Micro Combination pH Electrode with a glass body (dimensions: length, 150 mm; tip, 4.5 mm diameter × 90 mm length) were used to measure solution pH after irradiation.

As, over time, it is possible to observe small changes in electrode output, the pH electrode was calibrated every two weeks, ensuring accurate and reliable measurements. The electrode was inserted to a depth of approximately 80% of the container, and each prepared solution was measured twice. The pH probe was thoroughly rinsed with pure water and dried prior to each measurement. External interference was not present, and the ambient humidity, as it remained relatively stable, was not considered a significant factor.

It is noteworthy that DI water may initially have a pH close to neutral (~7), but it quickly decreases as it equilibrates with atmospheric CO₂. Additionally, because of its low ionic content, DI water is highly sensitive to external influences, such as minor environmental contaminants, which can further affect its pH, as seen in Figure 2 and Figure 3.

### 4.4. FTIR Measurements

To obtain the infrared spectra of our samples, we used a JASCO Fourier Transform Infrared Spectrometer (FT/IR 6800), Easton, MD, USA. Prior to scanning each sample, a background scan and a scan of a crystal without a sample were obtained. We placed 4 μL of the sample on the crystal, and after the sample was dry, we carried out measurements with 256 scans with a resolution of 4 cm^−1^ and a range of 600–4000 cm^−1^. The crystal scan was then subtracted from the sample scan to eliminate any possible contaminants.

## Data Availability

The data that support the findings of this study are available within the article and from the corresponding author upon reasonable request.
